# 16S rRNA gene amplicon sequencing data from the gut microbiota of adolescent Afghan refugees

**DOI:** 10.1016/j.dib.2024.110636

**Published:** 2024-06-17

**Authors:** Muhammad Shahzad, Anum Saeedullah, Muhammad Shabbir Khan, Habab Ali Ahmad, Ishawu Iddrissu, Simon C. Andrews

**Affiliations:** a Faculty of Dentistry, Zarqa University, Jordan; b Institute of Basic Medical Sciences, Khyber Medical University Peshawar, Pakistan; c Department of Biomedical Sciences, Pak-Austria Fachhochschule Institute of Applied Science and Technology (PAF-IAST), Haripur, Pakistan; d School of Biological Sciences, Health and Life Sciences Building, University of Reading, Reading, RG6 6EX, United Kingdom; e Prospect Park Hospital, Berkshire Healthcare NHS Foundation Trust, Reading, RG30 4EJ, UK

**Keywords:** Gut microbiome, Refugees, Adolescents, Nutritional status, Health

## Abstract

The gut microbiota residing in the distal ileum and colon is the most complex, diverse, and densest microbial ecosystem in the human body. Despite its known role in human health and disease, gut microbiome diversity and function are rarely explored in vulnerable populations such as refugees. The current study aimed to explore gut microbiota diversity and sources of variation among adolescent Afghan refugees residing in Peshawar, Pakistan. Stool samples were collected from 10 – 18 years old, healthy adolescents (n=205) for 16S rRNA gene sequence (V4-V5 hypervariable region) analysis on isolated faecal DNA. Bioinformatics analyses were performed using Kraken2, Bracken and Phyloseq. The data presented here will allow researchers to profile the gut microbiota of this rarely explored, vulnerable population who are at high risk of food insecurity and malnutrition. The data can be used to provide insight on the impact of demographic characteristics, dietary intake, nutritional status, and health on gut microbiome diversity, and enables a comparative analysis with similar data sets from other population groups of relevance. The amplicon sequencing data are deposited in the NCBI Sequence Read Archive as BioProject PRJNA1105775.

Specifications Table

**Table:** 

Subject	Microbiology.
Specific subject area	Metagenomic (Human Gut Microbiome)*.*
Type of data	Table, Raw, Analysed.
Data collection	Sociodemographic data and stool samples were collected from apparently healthy, adolescent (10 – 18 years old), Afghan refugees residing in Khazana refugee camp in Peshawar, Pakistan. Genomic DNA was extracted using a QIAmp Fast DNA Stool Mini Kit followed by 16S rRNA gene amplicon sequencing on an Illumina MiSeq platform
Data source location	The samples were collected in an Afghan refugee camp located in Peshawar, Pakistan. 34.0151° N, 71.5249° E)*.*
Data accessibility	Repository name: NCBI Sequence Read Archive (SRA)… Data identification number: BioProject PRJNA1105775 Direct URL to data: https://www.ncbi.nlm.nih.gov/sra/PRJNA1105775
Related research article	*None.*

## 1 Value of the Data

• The data provides the first comprehensive report on the gut microbiota composition of Afghan refugees, one of the most protracted refugee communities in the world.
• This dataset provides valuable insights into the relationship between dietary intake, nutritional status, and gut microbiota composition of this rarely explored population.
• The data provides a baseline for future research on the gut microbiome and health outcomes in refugee populations. This information can be used to monitor changes over time and evaluate the effectiveness of interventions aimed at improving health and nutrition in refugee population.
• The data can be used to develop targeted interventions to improve gut health and nutritional status in refugee populations.
• The future studies can use this data as reference to compare gut microbiome composition of other ethnicities in regions (e.g. Pakistan, Afghanistan) with similar cultures and dietary habits.

## 2 Background

The human gut microbiome, a complex and dynamic ecosystem of trillions of microorganisms residing in the digestive tract, plays a critical role in human health and disease. This diverse community of bacteria, archaea, fungi, and viruses interacts with the host immune system, influences nutrient metabolism, and contributes to the growth and development of the human host [1]. Research suggests that gut microbiome diversity and composition in humans is primarily influenced by factors such as age [2], geographic location [3], diet, nutrition [4], and individual health status [5]. However, gut microbiome diversity and associated sources of variations are rarely explored in vulnerable populations such as Afghan refugees, the second largest refugee communities in the world after Syria. Majority (around 2.4 million) of Afghan refugees reside in neighbouring country Pakistan [6]. Due to fragile economic conditions in the host country, Afghan refugee in Pakistan face a multitude of challenges including lack of, or poor, access to clean water, sanitation, shelter, and healthcare facilities [7]. Food insecurity and malnutrition (especially multiple micronutrient deficiencies) are also common [8]. The current study aimed to explore gut microbiome diversity and associated factors (demographics, dietary intake, nutritional status, and health) among adolescent Afghan refugees.

## 3 Data Description

**Fig. 1:**
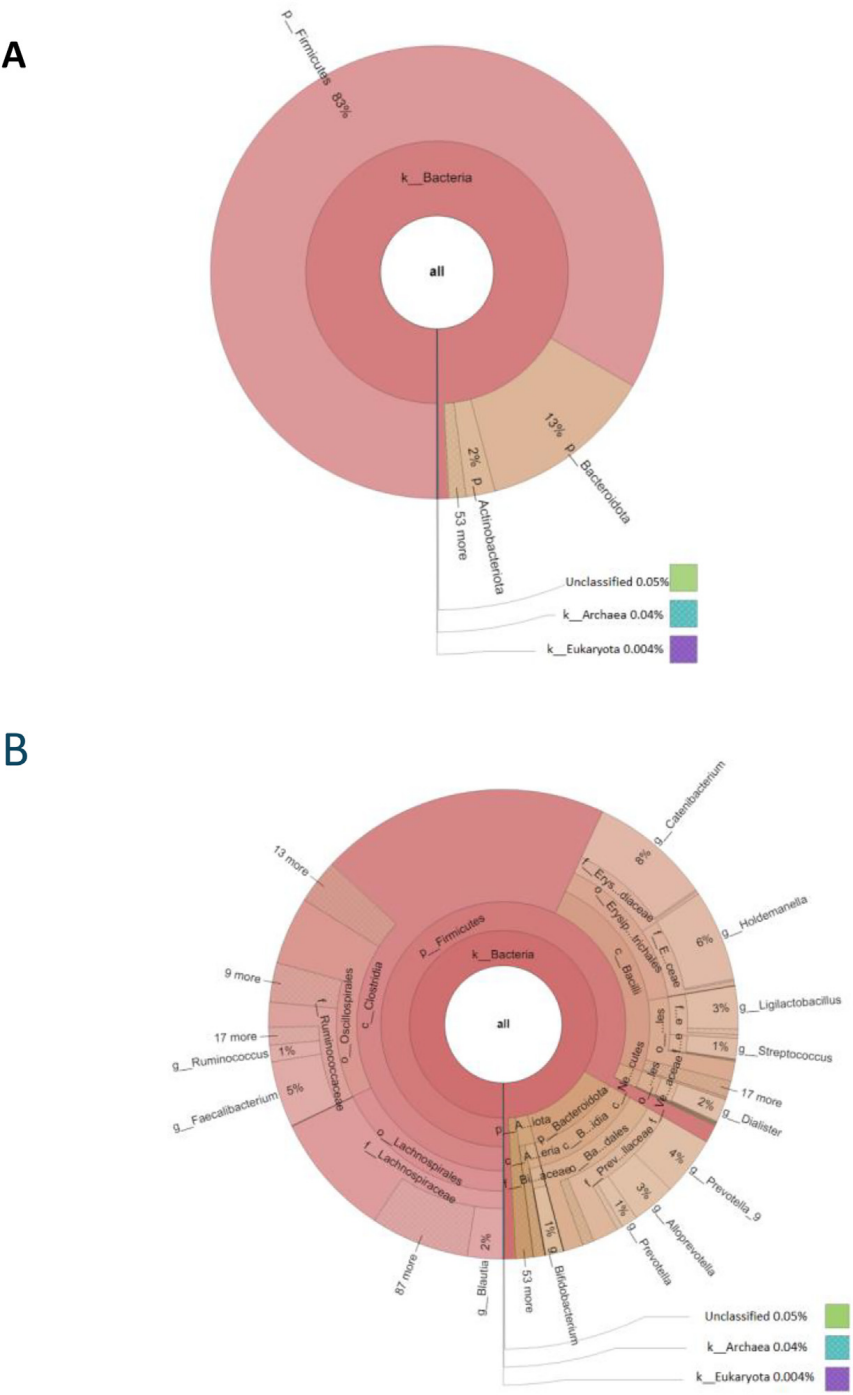
Korona charts showing gut microbiota diversity (a) Phylum level (b) Genus level.

The data presented here describe the gut microbiome composition of apparently healthy, adolescent Afghan refugees residing in a refugee camp in Peshawar, Pakistan. The latitude and longitude for the data collection site are located at 34.077585, 71.580361 respectively. DNA was extracted from fecal samples of 205 adolescents including 103 females and 102 males and were sent to the Animal and Plant Health Agency (Surrey, UK) for 16S rRNA gene sequencing. DNA was amplified with universal primers for the V4 and V5 regions of the 16S rRNA gene. Primers U515F (5'-GTGYCAGCMGCCGCGGTA) and U927R (5'-CCCGYCAATTCMTTTRAGT) [9] are designed to amplify bacterial and archaeal rRNA gene regions based on previously published literature [10]. The samples were sequenced using an Illumina MiSeq platform with paired-end runs. The raw sequencing reads were demultiplexed. As a result, two pair end fastq files were obtained for each sample. For example, for sample F001, the pair end fastq files are presented as F1_S1_R1_001.fastq.gz and F1_S1_R2_001.fastq.gz. The data set containing 16S rRNA amplicon raw sequences reads in pair end fastq format are deposited in the GenBank Sequence Read Archive under BioProject number PRJNA1105775. From the total number of 205 participants, those with low-quality stool-sample DNA sequence data (n=9) were excluded from all further analyses. After quality and taxonomy filtering, a total of 8750088 filtered reads were obtained by sequencing V3-V4 hypervariable region of 16S rRNA gene. The average number of reads per sample were 44643 ± 18857 (range: 14726 to 123047). Information about the individual sample's codes, gender, biosamples and SRA accession numbers and the number of raw, filtered and taxonomy assigned reads are presented in supplementary table 1. Taxonomic analysis reveals the presence of diverse gut microbiota belonging to 56 distinct phyla and 252 genera (Fig 1).

## 4 Experimental Design, Materials and Methods

### 4.1 Study design and setting

A randomized, community based, cross-sectional study design was employed. The study site was Khazana refugee camp; the largest Afghan Refugees Village (ARV) located in the outskirts of Peshawar city (34.077585, 71.580361), the capital of Khyber Pakhtunkhwa province of Pakistan. At the time of data collection (March–April 2020), the camp was home to more than 5000 Afghan refugees and 900 families.

### 4.2 Study population

A total of 205 adolescent children were recruited for the study. Sample size for the study was calculated using OpenEpi Epidemiologic Statistics software based on the following assumptions: (a) anticipated proportion of zinc deficiency in Afghanistan is 15.1% (NNS 2013); (b) absolute precision of 5%; (c) confidence level of 95%; (d) design effect of 1; and (e) a infinite population size. Inclusion criteria for recruited participants were: [1] adolescent boys and girls, 10 – 18 years old; [2] apparently healthy with no oral or systemic disease; and [3] not currently using or used antibiotics, nutritional supplements, probiotics or prebiotics, laxatives, antispasmodic or anti‐diarrhea drugs in the past month. Exclusion criteria included: residing outside the designated refugee village; those who were physically or mentally handicapped; and failure to provide a signature for the informed consent from.

### 4.3 Data and samples collection

Information about participant demographic and socioeconomic status were collected using a structured questionnaire. Dietary intake of the participants was assessed by trained nutritionist using 24-h dietary recall which was conducted through an in-depth, ∼25 minute interview using a standardized four stage protocol [11]. Details of all the food items and beverages consumed during the past 24-hour period were thus assembled. This included recording information about all food and beverages consumed, ingredients, cooking methods and brand names of commercial foods. The amount of each food or beverage consumed was estimated in reference to common size containers (bowls, cups and glasses), standard measuring cups and spoons and two dimensional aids (photographs). This information was collected from twice for each participant, on two different days of the week.

For collection of fecal samples, participants were provided with a stool sample collection kit one day prior to the sample collection date. The kit included a stool collection pot, a plastic bag and plastic gloves, and an instruction sheet explaining the sample collection procedure with a pictorial presentation of the process. The sample collection, packing and transport procedure was also explained verbally to all participants. On sample collection day, participants were instructed as follows: wear the gloves suppled; collect the stool sample directly into the collection pot; fill at least half of the screw-top tube with stool sample using the attached mini-spoon; tightly attach the lid to the tube; place the tube into the plastic bag; and final store the tube in the cool box before handing it over to the researcher. The participants were instructed to wash their hands with soap after collecting the stool sample and removing gloves. On reaching the field lab, aliquots of the samples (200 mg) were added into 1mL of DNA shield (Zymobiomics, USA) and stored at -80°C for further processing.

### 4.4 DNA extraction and 16S rRNA sequencing

Genomic bacterial DNA was extracted from the fecal samples already stored in DNA shield using a QIAamp® Fast DNA Stool Mini Kit (Qiagen, Valencia CA USA) following the manufacturer instructions. Each sample was quantified using a Nanodrop and integrity, quality and quantity were confirmed by agarose gel electrophoresis. DNA samples were then subjected to PCR to confirm suitability for 16S rRNA gene amplification. The samples were sequenced at a well-established Illumina MiSeq platform facility located at the Animal Health & Plant Agency (UK government sponsored agency). Sequencing involved PCR amplification of the V4-V5 hypervariable region of the 16S rRNA genes to give ∼400 bp amplicons. Concentrations for each sample were normalized and then samples were pooled (205 samples, with unique bar codes for each sample).

### 4.5 Bioinformatics analysis

The 16S rRNA gene paired end sequencing data were subjected to quality screening by removing chimeric sequences, dereplication of amplicons and determination of sequencing error rates using DADA2 software [12]. High quality sequencies (phred >30) were then obtained by trimming at reads at 280 and 160 bp for forward and reverse reads, respectively. To further ensure quality, the reads with N nucleotides and >2 expected errors were also discarded (maxN = 0, maxEE = 2, truncQ = 2). The final reads were then subjected to taxonomic profiling using the k-mer based tool Kraken2 (v2.1.2) and relative abundance estimation at different taxonomic levels usig Bracken (v2.8) [13]. Taxonomic classification and abundance estimation were performed using the Greengenes Database (v13.5) for Kraken2. Alpha diversity was assessed by read count data obtained from the Kraken2 analysis and the differences between different groups was compared using Wilcox test (significance <0.05). Relative abundance data obtained from Bracken were used to generate multidimensional scaling (MDS) plots for the comparison of β diversities based on Bray–Curtis dissimilarity matrices using the vegan package (v2.6.4). Linear discriminant analysis (LDA) effect size (LEfSe) analysis was performed with Phyloseq (v1.40.0). Phylogenetic Investigation of Communities by Reconstruction of Unobserved States (PICRUSt2 v2.5.2) software was employed for predicting the functional abundances based on marker gene sequences [14]. PICRUSt2 results were visualized with ggpicrust2 (v1.7.2) vignette [15]. All analyses were implemented in R version 4.2.3.

## Limitations

This study was limited by its cross-sectional design, which does not allow for causal inferences. Additionally, the sample size was relatively small, and the findings may not be generalizable to all refugee populations.

## Ethics Statement

The study protocol was approved by the Ethics Board of Khyber Medical University, Peshawar (DIR/KMU-EB/PR/000766). Informed consent was obtained from all participants or their parents/ legal guardians. Data confidentiality was maintained throughout the study.

## CRediT authorship contribution statement

**Muhammad Shahzad:** Conceptualization, Methodology, Formal analysis, Funding acquisition, Writing – original draft. **Anum Saeedullah:** Data curation, Formal analysis. **Muhammad Shabbir Khan:** Data curation, Formal analysis. **Habab Ali Ahmad:** Data curation, Writing – review & editing. **Ishawu Iddrissu:** Data curation. **Simon C. Andrews:** Supervision, Funding acquisition, Writing – review & editing.

## Data Availability

Gut microbiome diversity and composition of adolescent afghan refugees (Original data) (NCBI).

## Appendix Supplementary materials

Image, application 1
